# INFLUENCE OF NEGATIVE ATTITUDES TOWARD VOLUNTEER ACTIVITIES ON MENTAL HEALTH STATUS: THE REPRINTS STUDY

**DOI:** 10.1093/geroni/igad104.2872

**Published:** 2023-12-21

**Authors:** Yoshinori Fujiwara, Tomoya Takahashi, Hiroshi Murayama, Kyoko Fujihira, Koji Fujita, Mariko Yamashita, Hiroko Matsunaga, Hiroyuki Suzuki

**Affiliations:** Tokyo Metropolitan Institute for Geriatrics and Gerontology, Tokyo, Tokyo, Japan; Tokyo Metropolitan Institute for Geriatrics and Gerontology, Tokyo, Tokyo, Japan; Tokyo Metropolitan Institute of Gerontology, Tokyo, Tokyo, Japan; Tokyo Metropolitan Institute of Geriatrics and Gerontology, Tokyo, Tokyo, Japan; Tokyo Metropolitan Institute for Geriatrics and Gerontology, Tokyo, Tokyo, Japan; Tokyo Metropolitan Institute of Geriatrics and Gerontology, Tokyo, Tokyo, Japan; Tokyo Metropolitan Institute of Geriatrics and Gerontology, Tokyo, Tokyo, Japan; Tokyo Metropolitan Institute of Geriatrics and Gerontology, Tokyo, Tokyo, Japan

## Abstract

Although social engagement activities can help older adults maintain mental health status, whether negative attitudes toward volunteering influence poor mental health status due to psychological burdens has not been clarified. This study examined negative influence of intergenerational picture-book reading program “REPRINTS” on older volunteers’ attitudes to volunteering activities to clarify its impact on their mental health status. A questionnaire mail survey was conducted for 481 volunteers, ≥55 years, in 12 municipalities across Japan in 2020. Of the 423 respondents (87.9%), 266 responded to the follow-up survey, conducted from November 2021 to February 2022. Mental health status was assessed by WHO-5J; a score of less than 13 was regarded as poor mental health status. Multiple logistic regression analysis was performed on the responses of 220 women, aged 65–84 years, with WHO-5J (good ≥13 points; poor < 13 points) at follow-up as the dependent variable. Participants were asked about their positive and negative attitudes toward volunteer activities using a four-point scale (disagree to agree), and the scores were operationally summed up after converting them into a negative attitude score. Subjective health (healthy/unhealthy), age, WHO-5J score, and continuous years of activity at baseline were entered as adjustment variables. Sixty-four (29.1%) participants had a WHO-5J score of < 13 at baseline. Logistic regression analysis with WHO-5J as the dependent variable showed that the adjusted odds ratio for negative awareness scores was 1.06 (95% CI: 1.00-1.12). In conclusion, negative attitudes toward volunteer activities were suggested to have a significant influence on mental health status.

